# The Impact of Resource Bricolage on Entrepreneurial Orientation in Start-ups: The Moderating Roles of TMT Heterogeneity and TMT Behavioral Integration

**DOI:** 10.3389/fpsyg.2022.900177

**Published:** 2022-07-18

**Authors:** Peng Xiaobao, Guo Rui, Zu Jiewei, Song Xiaofan

**Affiliations:** School of Public Affairs, University of Science and Technology of China, Hefei, China

**Keywords:** start-ups, resource bricolage, entrepreneurial orientation, TMT heterogeneity, TMT behavioral integration

## Abstract

Prior studies demonstrate the role of resources in shaping a firm’s entrepreneurial orientation from the resource-based view. We expand this line of research by theorising and testing the impact of resource bricolage on entrepreneurial orientation. Based on the data of 295 start-ups, we find that when start-ups face resource constraints, the strategy of resource bricolage has a significant positive effect on entrepreneurial orientation, and the relationship is positively moderated by top management team (TMT) heterogeneity. Meanwhile, the relationship is negatively moderated by TMT behavioral integration. The results are expected to provide theoretical guidance for start-ups to overcome resource constraints and achieve smooth survival and growth.

## Introduction

Start-ups usually face a higher risk of entrepreneurial failure than mature enterprises. Start-ups face high levels of technological and market uncertainty due to liability or weakness caused by newness (Elfring and Hulsink, 2003). The lack of performance records and information asymmetry hinders the evaluation of resource owners, making it difficult for start-ups to obtain external resources (Xiumei and Yupeng, 2010). Previous studies have shown that cultivating entrepreneurial orientation can effectively promote dynamic capability orientation and firm performance and enterprise development (Wiklund and Shepherd, 2003) in uncertain environment. It can be seen that entrepreneurship orientation is very important for start-ups. Entrepreneurial orientation is a mental model of firms in pursuit of new business and in response to environmental change (Srivastava and Lee, 2005), Firms with a high entrepreneurial orientation are more likely to innovate continuously and actively defeat competitors perceived as more entrepreneurial (Miller, 1983; Anderson et al., 2015). There are few studies on the antecedent variables of entrepreneurship orientation. Although researchers have recognized the importance of resources for entrepreneurial orientation and established a framework involving firms’ internal resources, competitive advantages and innovation capability, most research efforts of entrepreneurial orientation are limited to the entrepreneurial resources themselves. Therefore, these studies cannot yet provide an effective answer to the problem of resource scarcity (Jingkun and Jian, 2019).

The key to the survival and development of start-ups facing fierce market competition lies in their creative reorganization of resources (Sirmon et al., 2011). Resources are the foundation of entrepreneurial activity (Barney, 1991). When firms have the resources to match their (Miller, 1983) entrepreneurial behavior and innovation, they tend to succeed. Most firms face massive resource constraints (Wiklund and Shepherd, 2005), while start-ups face even more severe resource constraints. Resource bricolage is an action strategy for entrepreneurs to meet new entrepreneurial opportunities or challenges by adapting and leveraging existing resources (Baker and Nelson, 2005). Successful bricolage enables start-ups to cope better with market uncertainty, survive in resource constraints, and perhaps even thrive (Lomberg et al., 2017). How does bricolage affect entrepreneurial orientation? The mechanism remains to be explored.

The other key to deepening research on the relationship between resource bricolage and entrepreneurial orientation lies in the introduction of related organizational factors. Entrepreneurial behavior is rooted in a certain resource environment, and TMT of each firm is the decision-maker and executor of the corporate strategy and plays a key role in the enterprise innovation activities (Hambrick and Mason, 1984). The theoretical extension based on the upper echelons theory proves that the characteristics of TMT (such as age, career path, other professional experience, education, socioeconomic basis, economic status, group characteristics, etc.) are important organizational factors that affect entrepreneurial orientation (Bantel and Jackson, 1989; Yang and Wang, 2014). However, there are still considerable differences in the empirical research conclusions on the relationship between TMT and entrepreneurial orientation. Start-ups usually face unstructured and creative problems, and heterogeneous teams are often better able to cope with them due to their different cognitive and resource bases (Gang and Chao, 2017). TMT behavioral integration is defined as the degree of team members’ participation and interaction in thought and action, which holds the key to the effectiveness of strategic decision-making and execution (Carmeli and Schaubroeck, 2006). The demographic differences of TMT members and their behavioral integration influence the decision-making and implementation of corporate strategy, which can explain the group phenomenon (Alexiev et al., 2010).

Our study focuses on the impact of resource bricolage on entrepreneurial orientation in start-ups, effectively combining the entrepreneurial resource view and entrepreneurial opportunity view to provide a new entrepreneurial research perspective. That is, when start-ups cannot control all the resources, bricolage behavior helps the entrepreneur use the existing resources optimally and avoid resource restrictions. Therefore, resource bricolage is an effective solution to overcome the resource-constraint dilemma, providing an effective way for enterprises to carry out entrepreneurial activities. In addition, resource bricolage results from TMT’s decisions, so it does not independently affect entrepreneurial orientation. Analysis of this TMT dual role can explain why some start-ups with similar resource bases succeed and others fail. By considering this view, we provide a new way of thinking and a feasible path for start-ups to overcome the resource dilemma (Mehrabi et al., 2021). It also deepens the research content of the perspective of resource bricolage and upper echelons theory.

## Theory and Hypotheses

The positive effect of entrepreneurial orientation on performance through various ways has been gradually confirmed (Li et al., 2009; Lomberg et al., 2017). Thus, entrepreneurs and scholars began to focus on what can be done to make the most of entrepreneurial orientation (Wangbin and Yuli, 2012). Following the conceptual structure of entrepreneurial orientation proposed by Lumpkin and Dess (1996), scholars study the impact of environmental factors, strategic factors, and internal factors on entrepreneurial orientation. The entrepreneurial behavior of firms is regarded as a process of integrating internal and external resources. Therefore, resources are the foundation of entrepreneurial activity and the key to its success (Barney, 1991). The quantity and quality of resources play a key role in the performance, survival, and development of entrepreneurial firms (Guohong and Lan, 2018).

Prior studies focused on the important role of resources for firms to build and maintain strategic advantages from the resource-based view (Covin et al., 2006; Jian, 2012), and most concluded that competitive advantage and high performance of firms come from a unique and heterogeneous resource portfolio (Wales et al., 2011). The view highlights that advantageous resources must have three characteristics simultaneously: they can create value, cannot be copied by competitors, and can be continuously possessed. The resource-based view discusses the source of competitive advantage from the perspective of internal enterprise resources, but scholars gradually realized that the formation and accumulation of competitive resources is a long-term process (Shijian and Minghui, 2013). It is difficult for start-ups to achieve this in the early stages. Moreover, the view does not extend to research on how to obtain advantageous resources and the abilities needed by firms to obtain resources.

When it is difficult for start-ups to obtain advantageous resources or they even face the problem of insufficient resources, integrating and utilizing existing resources are effective ways to address the problem (Yuli and Xin, 2009). The problem is acute for start-ups, which typically need many resources in the start-up and growth stages. Scarcity is the most striking feature of entrepreneurial resources (Liang and Heng, 2017). Due to the lack of internal accumulation, such a firm often does not have all the resources needed to develop its opportunities (Senyard et al., 2014). Therefore, one of the important tasks of entrepreneurs in the entrepreneurial process is to access and utilize resources (Newbert and Tomikoski, 2012). The concept of resource bricolage, proposed by Baker and Nelson (2005), provides a new thinking style and a new action strategy to solve resource problems. Following the principle of “make the best use of everything,” firms make do with their existing, accessible resources to explore opportunities and meet challenges. When start-ups are unable to pay for standardized resources that have a high degree of matching needs, strong applicability, and high efficiency, the original purpose can be achieved by using the resources currently at hand (Hongxia and Hongjia, 2016).

### Resource Bricolage and Entrepreneurial Orientation

Scholars proposed the logic of potential advantages of resource constraints, that is, the effects of resource constraints are not all negative (Gibbert et al., 2007). The more severe the resource constraint of a start-up, the more efficient the start-up will be in using resources. They tend to work harder to find resources in competitive markets, partly reflecting greater entrepreneurial intention. Resource bricolage includes three core elements, namely the resource at hand, making do, and combining resources for new purposes (Miller, 1983). The first element, “resource at hand,” relates to entrepreneurial orientation because firms pay attention to the use and exploration of resources that are immediately available, especially resources that exist in new firms or the existing market but have not been explored or neglected (Covin and Slevin, 1989). Using existing idle resources, which not only reduces resource costs and saves search time, but also obtains income from investment, which greatly improves the ability of firms to take entrepreneurial risks (Xiue and Kun, 2018). Similarly, through the creative use of inexpensive resources, firms can bring more net cash inflow with minimal cost, achieving survival and development (Hooi et al., 2016). In addition, firms may inadvertently create new resources by using many piecemeal resources; the process helps firms market new products, provide new services, and achieve innovation (Lu et al., 2019).

The second core element listed above, “making do,” relates to entrepreneurial orientation as follows. This form of bricolage refers to the effective performance of an entrepreneur who faces resource constraints and uses existing resources to deal with new challenges or opportunities (Baker and Nelson, 2005). On the one hand, “making do” helps firms creatively solve the problems they face and quickly create targeted products, services, or business models, thereby making more room for the firms to develop (Jingqin and Jingjing, 2017). On the other hand, “making do” is advantageous to seize the fleeting opportunity to take the lead in breaking into new markets and thus gain a first-mover advantage (Zahra and Covin, 1995).

The third core element, “combination of resources for new purposes,” relates to entrepreneurial orientation because entrepreneurs frequently integrate resources that were previously used for other purposes to achieve new goals (Liang and Xinglu, 2016). It means that start-ups often use resources that were otherwise used for other purposes to achieve goals and capture market opportunities. This is an extension of the use of resources, and is an important manifestation of bricolage (Desa, 2012). The new service attribute is developed by the firm based on the original use attribute of the resource, which helps the firm to get out of the difficulty of obtaining the standard resource in time, and to simultaneously improve stability in the existence and development of the organization.

Resource bricolage is an important way for firms to obtain available resources by exerting their subjective initiative. Although the literature contains no direct research showing that resource bricolage impacts the entrepreneurial orientation of enterprises, scholars have verified the relationship between resource bricolage and innovation performance through empirical research, which reflects the innovation and risk-taking ability of enterprises. Based on this, we consider the following hypothesis:

*H1*: A firm’s resource bricolage positively relates to its entrepreneurial orientation.

### The Moderating Effect of Top Management Team Characteristics

When studying resource strategies, one should not neglect examination of the subject of decision-making, that is, TMT. According to the upper echelons theory, there are two main viewpoints (Hambrick and Mason, 1984). Firstly, when faced with the same organizational environment and strategic information, different managers make different strategic choices and interpret the information diversely (Carpenter et al., 2004). Scholars have studied the impact of entrepreneurial enthusiasm on entrepreneurial behavior and received positive responses (Li et al., 2020). Secondly, the differences arise from the past experiences, values, perceptions, and personal characteristics of the top managers. Based on the above two main points, Hambrick and Mason further suggest that to understand why a firm makes one choice rather than another, it is necessary to have a deep understanding of its top managers. The theory effectively explains why enterprises with similar resource constraints may have completely different survival and growth capabilities (Knight et al., 2015).

The TMT background characteristics are closely related to key activities such as the formulation and execution of strategic decisions (Xinming and Huan, 2021) because a TMT is usually made up of key managers responsible for strategy formulation, planning, and implementation. They are responsible for the operation and management of the entire organization and have decision-making and control rights (Hambrick, 2007). Therefore, the TMT decision-making process will impact the firm resource allocation and change. Therefore, considering the integrity of the research model, the current study combines TMT heterogeneity and TMT behavioral integration into the same research framework. The theoretical model can further deepen and expand the upper echelons theory and the perspective of resource bricolage.

### The Moderating Effect of Top Management Team Heterogeneity

TMT heterogeneity reflects the differences of TMT in demographic characteristics, important cognitions, values, and experiences, which can be divided into demographic background variables and latent variables (Mehrabi et al., 2021). Often, heterogeneous teams are better suited to deal with unstructured, creative problems (Alexiev et al., 2010). Therefore, when the diversity among TMT members is large, the team has diversified knowledge, skills, experience. Moreover, diversity means that the team has a wide social network and a broad interpersonal base, which can provide more diverse resources and capabilities for the growth of the venture (Heyden et al., 2013). Conversely, when the level of TMT heterogeneity is low, it is often difficult to have sufficient external contacts to access strategic resources.

The background characteristics of top managers determine their problem-solving ways and thus influence their strategic decisions (Richard et al., 2019). Therefore, the influence of TMT characteristics in the study of the relationship between resource bricolage and entrepreneurial orientation should not be ignored. On the one hand, highly heterogeneous teams have a wide variety of information and insights and a broader perspective, which allows them to identify more potentially available resources (Bantel and Jackson, 1989). When the use of resources is broadened, the team’s ability to solve problems is enhanced. On the other hand, high heterogeneity means that the team has more extensive social capital and network relationships, which can improve the firm’s ability to access both tangible and intangible resources. For both reasons, TMT member heterogeneity is helpful for start-up firms to cope with resource constraints and achieve high growth. Based on this, the following hypothesis is made:

*H2*: TMT heterogeneity positively moderates the relationship between resource bricolage and entrepreneurial orientation; the higher the degree of TMT heterogeneity, the greater the positive impact of resource bricolage on entrepreneurial orientation.

### The Moderating Effect of Top Management Team Behavioral Integration

Based on the upper echelons theory, Hambrick found through field research that it is not enough to rely on static indicators such as demographic characteristics to predict the results of firms, and TMT behavioral interaction are also important. If TMT members cannot effectively integrate their existing knowledge and skills, it is often difficult to find new opportunities. The solidification of knowledge and experience is easy to form cognitive bias, which will lead to the solidification of the existing strategy and affect the innovation behavior of the enterprise. In 1994, the concept of “TMT behavioral integration” was first put forward and the term can be used to describe the essence of the specific operation process of the team. As a meta-concept of the TMT executive process, TMT behavioral integration is the process of TMT members sharing information, resources, and decision; these three aspects reflect the team’s integrating capacity.

After 1994, follow-up study based on the upper echelons theory showed that the integration of group behavior is an important contingent factor that influences the strategic behavior of firms (Hambrick, 2007), and thus impacts the relationship between resource bricolage and entrepreneurial orientation. For two reasons, this study suggests that TMT behavioral integration may weaken the positive relationship between resource bricolage and entrepreneurial orientation. Firstly, a high level of TMT behavioral integration indicates frequent information exchange among team members, but this often leads to the disadvantage that the members have the same decision-making basis, so they have similar access to resources (Simsek et al., 2005). In such a situation, it is difficult to use resources creatively. In contrast, a low level of behavioral integration means that team members’ unique vision of how to acquire resources can play a positive role. Second, joint decision-making is an important criterion for TMT behavioral integration. However, joint decision-making is often based on risk reduction, so teams tend to form a conservative consensus. Such a team ignores abandoned or idle resources, which is not conducive to the discovery, utilization, and accumulation of resources. Based on this, the following hypothesis is made:

*H3*: TMT behavioral integration negatively moderates the relationship between resource bricolage and entrepreneurial orientation; the higher the degree of TMT behavioral integration, the weaker the positive effect of resource bricolage on entrepreneurial orientation.

## Materials and Methods

### Sample and Data Collection

A questionnaire survey was employed to collect data to test our hypotheses. Since this study focuses on firms in the early stages of formation or growth, we use the standards within the Global Entrepreneurship Monitor (GEM) report to investigate firms that have been established for at most 42 months. Considering that the CEO and senior management team members are located at the top of the organizational structure, they play an active role in the strategic decision-making of the enterprise. Meanwhile, they are familiar with the overall operation of the enterprise and can more accurately reflect the real situation (Yong and Rui, 2019). We first sent a questionnaire to CEOs to measure their perceptions of entrepreneurial orientation. At the same time, we sent a questionnaire to the top management team members to gauge their views on the firm’s resource bricolage. To improve the response rate, our questionnaire was distributed by a professional team in China, a leader in Chinese market research. Specifically, the team uses a data platform dedicated to providing large-scale research, data collection, modeling, analysis, and business applications solutions for research institutions, businesses, and individuals.

A small-scale sample survey confirmed the reliability and factor structure of our measurements. Then, with a good understanding of our research purpose and requirements, the group recruited respondents in a rigorous manner to form a high-quality, representative sample. Samples came from 21 provinces, autonomous regions, and municipalities directly under the central government in China, covering the four major economic regions: the northeast region (e.g., Heilongjiang Province, Jilin Province and Liaoning Province), the eastern region (e.g., Zhejiang Province, Shanghai, Jiangsu Province), the central region (e.g., Anhui Province, Shanxi Province, Jiangxi Province) and the west area (e.g., Chongqing, Sichuan Province, Guangxi).

We chose to conduct our research in China for two main reasons. On the one hand, with the concept of “Mass entrepreneurship and innovation,” China has formed a new wave of entrepreneurship (Jun and Yuli, 2020). As a result, the number of start-ups is huge, and research on entrepreneurship is of great significance to the country. However, as a developing country, China is at a disadvantage in the international transfer of resources, so it needs to focus on internal and limited resources. On the other hand, as the world’s second-largest economy, China’s enterprise development faces a severe domestic and international environment. Therefore, Chinese companies must mold a competitive advantage in a fierce market environment, and the role of their top management teams cannot be underestimated. Top managers are the right people to fill out the questionnaire, this happens because we need to consider the TMT characteristics in our study. More importantly, top managers are at the top of the organizational structure, playing an active role in making strategic decisions, and they are very familiar with the overall operating situation of the firm.

### Common Method Bias

In this study, we avoid the influence of common method bias by means of program control. Firstly, in the questionnaire design phase, we changed the order of items while keeping the same basic information to avoid reflecting bias. At the same time, the questionnaire uses a reverse item and a repeated item to identify invalid samples, which can help us quickly check whether the interviewee answered the question seriously. Secondly, before the respondents filled out the questionnaire, we informed them that the responses would be anonymous and the data would be used for scientific research only; all response information would be kept strictly confidential so it would not affect their work in any way. The team used IP address checking to ensure that each person could only answer once, and we ended up with 350 complete samples. Finally，by comparing the results of the polygraph item and the repeated item, the data of those who did not answer seriously were deleted, leaving 295 samples and an effective recovery rate of 84.29%. Table 1 shows the demographic details of the 295 respondents.

**Table 1:** Sample feature distribution (*N* = 295).

	Item	*N*	Percentage		Item	*N*	Percentage
				Major work experience	finance	77	26.55
			marketing		26	8.81
Gender	Male	184	62.37		manufacturing	17	5.76
	Female	111	37.63		technology	36	12.20
					administration	68	23.05
law	30	10.17		
			other		41	13.90
Age	≤30	127	43.05	TMT Numbers	≤5	27	9.15
	31–40	152	51.53		6–10	145	49.15
	>40	16	5.42		>10	123	41.70
				Firm Scale	≤50	17	5.76
Education	Junior college or below	34	11.53		51–100	43	14.58
	Bachelor	206	69.83		101–250	54	18.31
	Master degree or above	55	18.64		251–500	68	23.05
					501–1,000	61	20.68
					>1,000	52	17.63

### Measures

We adopted mature scales from the literature to measure, and we ensured the equivalence of language through translation and back-translation. Firstly, we translated the original scale from English into Chinese, and then translated the Chinese scale into English. Then, we invited two native English speakers to check the translated English scale. We repeated the operation until the new scale was consistent with the original scale in content, semantics, format, and application, after which we regarded it as a valid scale for distribution. After team discussion and feedback, we revised several ambiguous items and formed the final questionnaire. In addition to the control variables, all items were measured with 5-point Likert scales, ranging from “Strongly disagree” (1) to “Strongly agree” (5).

Resource Bricolage. We measured resource bricolage using eight items adapted from the work of Senyard et al. (2014), which includes three dimensions. Resources at hand refers to resources that exist in the market but have not yet been found or exploited for alternative uses; such resources are often obtained at a lower cost than standard alternatives. Making do means that the firm is quick to seize opportunities based on satisfaction rather than optimization. Combination of resources for new purposes refers to the reorganization of resources in order to achieve new goals. The internal consistency of resource bricolage was 0.828. The results of confirmatory factor analysis (CFA) show that the data represents the true characteristics of the measured objects (*X*^2^ = 33.324, *df* = 20, *X*^2^*/df* = 1.666, *p* = 0.031, RMSEA = 0.048[0.014, 0.075], GFI = 0.973, NFI = 0.948, IFI = 0.978, TLI = 0.969, CFI = 0.978). These results show that the scale had good aggregation validity.

TMT heterogeneity. We measured TMT heterogeneity using four items adapted from the work of Heyden et al. (2013). The scale measures professional knowledge field, experience, functional background, and complementarity. The internal consistency of TMT heterogeneity is 0.644, and the results of CFA are as follows: *X*^2^ = 14.876, *df* = 2, *X*^2^*/df* = 7.438, *p* = 0.001, GFI = 0.978, NFI = 0.902, IFI = 0.914, CFI = 0.911. These results indicate that the aggregation validity of the scale is good.

TMT behavioral integration. We measured TMT behavioral integration using nine items adapted from the work of Simsek et al. (2005), which include three dimensions. *Information exchange* reflects the initiative consciousness and the importance of the information exchanged in decision-making. *Collaborative behavior* measures how much the top managers work together and whether the boundaries of their rights and responsibilities are clear. Whether the firm’s important decisions are made through inter-team discussion is mainly used to measure *joint decision-making*. The internal consistency of TMT behavioral integration is 0.795 and the results of CFA are as follows: *X*^2^ = 41.107, *df* = 27, *X*^2^*/df* = 1.522, *p* = 0.040, RMSEA = 0.042[0.009, 0.067], GFI = 0.969, NFI = 0.931, IFI = 0.975, TLI = 0.966, CFI =0.975. These results show that the aggregation validity of the scale is excellent.

Entrepreneurial orientation. We use the scale developed by Covin and Slevin (1989), which includes three dimensions, with a total of nine items. Innovativeness refers to the firm having new ideas in terms of products, service, and technology. For example, since the firm was established, new products and new services have been developed, and there is a trend of sustainable development. Risk taking refers to the firm daring to face, undertake, or engage in behavior with a certain amount of danger; that is, the firm is more inclined to try rather than give up in the face of an uncertain environment. Proactiveness refers to the tendency of the firm to develop and market new products and services before other firms in the industry. The internal consistency of entrepreneurial orientation is 0.845. The results of CFA are as follows: *X*^2^ = 80.791, *df* = 27, *X*^2^*/df* = 2.992, *p* = 0.000, GFI = 0.941, NFI = 0.902, IFI = 0.933, TLI = 0.909, CFI = 0.932. These results show that the scale has good convergent validity.

Control variables. This study selects several variables which may affect the entrepreneurial orientation from the entrepreneur, the top management team and the start-ups level. Firstly, we control the gender of entrepreneur, because it makes differences in the degree of entrepreneurial inclination. According to the questionnaire, the entrepreneur’s age and education level were controlled by the ordinal classification variables. Different work experience results in different entrepreneurial intention, therefore, the main work experience is divided into seven categories, such as finance and accounting, marketing, production and manufacturing, technology research and development, administration, Discipline inspection and law. Second, from the perspective of entrepreneurial team, the number of team to measure the size of the TMT. Finally, the start-up scale is measured by the total number of enterprises at the enterprise level.

## Results

### Descriptive Statistics and Correlation Analysis

Table 2 presents the means, standard deviations, and correlations. The results show that resource bricolage correlates positively with entrepreneurial orientation (*r* = 0.71, *p* < 0.01). Resource bricolage also correlates positively with TMT heterogeneity (*r* = 0.61, *p* < 0.01), and TMT heterogeneity correlates positively with entrepreneurial orientation (*r* = 0.59, *p* < 0.01). Resource bricolage also correlates positively with TMT behavioral integration (*r* = 0.65, *p* < 0.01), and TMT behavioral integration correlates positively with entrepreneurial orientation (*r* = 0.71, *p* < 0.01).

**Table 2:** Descriptive statistics and correlation coefficients.

S. No.	Variables	*M*	*SD*	1	2	3	4	5	6	7	8	9	10
1.	Resource bricolage	4.23	0.45	—									
2.	TMT heterogeneity	4.29	0.45	0.61^**^	—								
3.	TMT behavioral integration	4.14	0.45	0.65^**^	0.64^**^	—							
4.	EO	4.05	0.53	0.71^**^	0.59^**^	0.71^**^	—						
5.	Gender	1.38	0.49	0.00	0.07	0.02	0.00	—					
6.	Age	1.63	0.61	0.01	0.05	−0.03	−0.05	−0.15	—				
7.	Education	2.07	0.55	−0.02	−0.02	0.00	0.05	−0.06	−0.05	—			
8.	Work experience	1.16	0.37	−0.06	−0.14	−0.06	−0.06	−0.03	0.04	−0.02	—		
9.	TMT Numbers	2.33	0.64	−0.05	−0.06	−0.09	−0.06	0.10	0.00	−0.11	0.00	—	
10.	Firm Scale	3.91	1.48	0.02	0.05	−0.04	0.01	−0.08	−0.03	0.10	−0.63	0.00	—

*Gender, 1 = male, 2 = female*.

** *p < 0.01*.

Variance inflation factor (VIF) test was carried out in this study, and the result was 1.025, close to 1, indicating that multicollinearity is not serious. In order to ensure the reliability of regression results and reduce statistical errors, this study conducted mean-centered processing on data before regression analysis.

### Hypothesis Testing

#### Main Effect Test

First, the regression analysis showed that resource bricolage has a significant positive effect on entrepreneurial orientation (*β* = 0.83, *SE* = 0.05, *p* < 0.001). Hypothesis 1 is verified.

#### Moderating Effect Test

The PROCESS 3.5 procedure (Model 1) of SPSS23.0 was used to test the moderating effect of TMT heterogeneity. The second hypothesized moderator was tested similarly. Table 3 shows that the moderating effect of TMT heterogeneity on the relationship between resource bricolage and entrepreneurial orientation was not significant (*β* = 0.04, *t* = 0.03, 95% CI [−0.025, 0.110]). Moreover, Table 4 examines the moderating effect of behavioral integration on the relationship between resource bricolage and entrepreneurial orientation was not significant (*β* = −0.03, *t* = −0.03, 95% CI [−0.084, 0.038]).

**Table 3:** The moderating effect of TMT heterogeneity.

Regression equation		Significance of overall equation		Significance of regression coefficient	
Outcome variable	Predictor variables	*R* ^2^	*F*	*β*	95%CI
EO	Constant	0.74	39.84^***^	0.02	[−0.761, 0.797]
	Resource bricolage			0.56^***^	[0.458, 0.656]
	TMT heterogeneity			0.27^***^	[0.173, 0.375]
	Resource bricolage ^*^ TMT heterogeneity			0.04	[−0.025, 0.110]
	Gender			−0.05	[−0.220, 0.110]
	Age			−0.11	[−0.240, 0.020]
	Education			0.12	[−0.026, 0.262]
	Work experience			0.03	[−0.253, 0.303]
	TMT Numbers			−0.02	[−0.142, 0.105]
	Firm Scale			−0.01	[−0.075, 0.064]

*** *p < 0.001*.

**Table 4:** The moderating effect of TMT behavioral integration.

Regression equation		Significance of overall equation		Significance of regression coefficient	
Outcome variable	Predictor variables	*R* ^2^	*F*	*β*	95%CI
EO	constant	0.78	92.42^***^	−0.15	[−0.874, 0.567]
	Resource bricolage			0.43^***^	[0.338, 0.526]
	Behavioral integration			0.43^***^	[0.330, 0.522]
	Resource bricolage ^*^ behavioral integration			−0.02	[−0.084, 0.038]
	Gender			−0.02	[−0.176, 0.128]
	Age			−0.06	[−0.178, 0.062]
	Education			0.10	[−0.033, 0.235]
	Work experience			0.02	[−0.235, 0.277]
	TMT Numbers			0.01	[−0.110, 0.120]
	Firm Scale			0.01	[−0.051, 0.077]

*** *p < 0.001*.

Finally, we examine the dual-moderating effects of TMT heterogeneity and TMT behavioral integration. Using Model 2 in the PROCESS 3.5 plug-in, we obtain the results shown in Table 5. The moderating effect analysis showed that TMT heterogeneity had a significant moderating effect on the relationship between resource bricolage and entrepreneurial orientation (*β* = 0.08, *t* = 2.01, *p* < 0.05, 95% CI [0.002, 0.166]), which was positive. Under the same conditions, the relationship between resource bricolage and entrepreneurial orientation was significantly mediated by TMT behavioral integration (*β* = −0.09, *t* = −2.01, *p* < 0.05, 95% CI [−0.161, −0.002]), which was negative. Thus, Hypotheses 2 and 3 are verified.

**Table 5:** Double regulation effect.

Regression equation		Significance of overall equation		Significance of regression coefficient	
Outcome variable	Predictor variables	*R* ^2^	*F*	*β*	95%CI
EO	Constant	0.79	44.14^***^	−0.20	[−0.918, 0.514]
	Resource bricolage			0.40^***^	[0.299, 0.498]
	TMT heterogeneity			0.14^**^	[0.040, 0.249]
	Resource bricolage ^*^ TMT heterogeneity			0.08^*^	[0.002, 0.166]
	Behavioral integration			0.35^***^	[0.244, 0.459]
	Resource bricolage ^*^ behavioral integration			−0.09^*^	[−0.161, −0.002]
	Gender			−0.05	[−0.202, 0.100]
	Age			−0.07	[−0.184, 0.054]
	Education			0.09	[−0.033, 0.232]
	Work experience			0.01	[−0.191, 0.200]
	TMT Numbers			0.15	[−0.099, 0.129]
	Firm Scale			0.02	[−0.046, 0.082]

* *p* *< 0.05;*

** *p* *< 0.01;*

*** *p < 0.001*.

**Figure 1:**
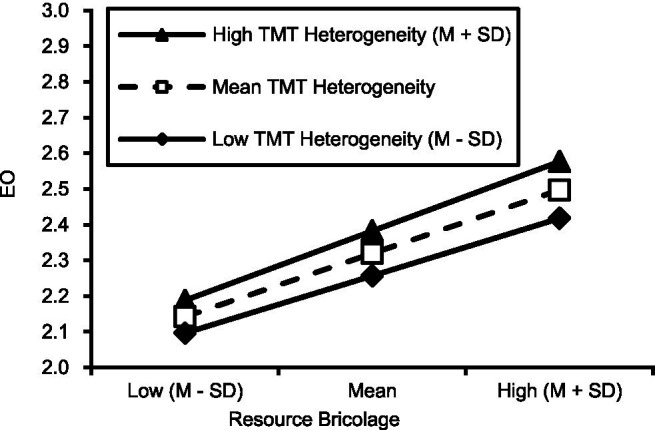
TMT heterogeneity’s moderating effect.

**Figure 2:**
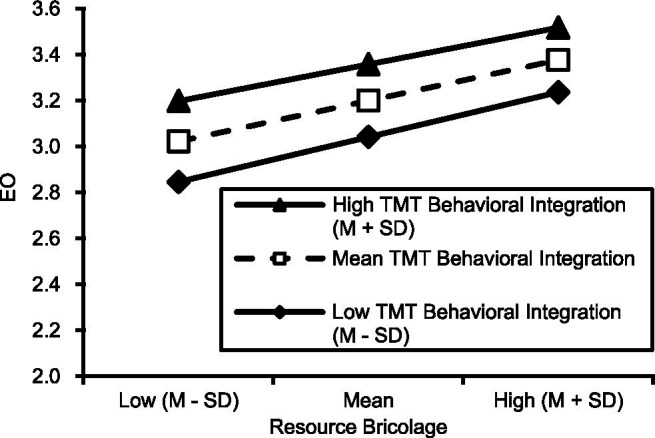
TMT behavioral integration’s moderating effect.

To explore the moderating effect of different degrees of heterogeneity, the resource bricolage and heterogeneity results were divided into three groups according to the average value plus or minus one standard deviation. Through the analysis of the following data and make moderating effect slope chart in Figure 1. The TMT behavioral integration is shown in Figure 2.

The simple slope is the most significant when the heterogeneity value is 1.02, and the behavioral integration value is −0.81. In other words, under the effect of high heterogeneity and low behavioral integration, the resource bricolage can influence entrepreneurial orientation positively, which can promote entrepreneurial orientation.

## Discussion and Conclusion

Based on resource bricolage theory, this study proposes and tests the impact model of 295 start-ups’ resource bricolage on entrepreneurial orientation. Previous studies have verified that resource bricolage has a positive impact on enterprise innovation and other outcome variables (Desa and Basu, 2013; Senyard et al., 2014), and also verified that resource integration positively influences firm entrepreneurship through innovation capability (Ling et al., 2020). However, there are no direct research shows that resource bricolage has an impact on entrepreneurial orientation. This study fully considers the resource environment of start-ups and expands the result effect of resource bricolage. We found that the higher the degree of resource bricolage, the stronger the entrepreneurial orientation of start-ups.

As the study showed, start-ups often face the dilemma of resource constraints and the need for innovation, which forces the firm to make full use of existing resources to create value and build capability (Zhenduo and Xinchun, 2016). This view provides a good explanation of why some start-ups stand firm in the entrepreneurial wave and bear market risks effectively: their success stems mainly from their reorganization and utilization of existing resources. When start-ups have more bricolage behaviors, they can use existing low-cost resources to provide more resource options through a quick assessment of the market environment, thus improving their ability to withstand risk, and the combination of new options is more conducive to enterprise innovation. The study has shown to some extent that resource bricolage constitutes an important base on which new firms can implement their entrepreneurship orientation. Start-ups need to pay attention to the accumulation and effective use of existing resources, strive to maintain flexibility in their problem solving, and lay a solid foundation for their entrepreneurship orientation and subsequent entrepreneurial activities from the perspective of resource bricolage.

Based on upper echelons theory, the current paper considers TMT heterogeneity and TMT behavioral integration from static and dynamic dimensions to explore the moderating effect between resource bricolage and entrepreneurial orientation. In terms of entrepreneurial team construction, most scholars study the impact of TMT heterogeneity on enterprise innovation, enterprise growth, resource acquisition and performance (Chowdhury, 2005; Zhou and Rosini, 2015) by upper echelons theory. Previous studies focused on the impact of TMT behavioral integration on entrepreneurial performance and firm innovation (Baoshan and Zhaorui, 2019), but did not form a unified opinion. This paper builds a research framework based on start-ups, and incorporates the resource pooling and entrepreneurial orientation of enterprises into the research, which is an important supplement to existing research. The study shows that TMT Heterogeneity and TMT Behavioral Integration have a significant dual-moderating effect on the relationship between resource bricolage and entrepreneurial orientation. That is, TMT heterogeneity strengthens the positive relationship between resource bricolage and entrepreneurial orientation, while TMT behavioral integration weakens the positive relationship between resource bricolage and entrepreneurial orientation. Therefore, when the degree of TMT heterogeneity is high and the degree of TMT behavioral integration is low, resource bricolage can significantly enhance entrepreneurial orientation.

Among the key driving factors for organizational innovation, the differences in age, knowledge, and abilities of top management team members often affect the generation of innovative ideas and the implementation of innovative behaviors, which in turn affect decision-making results (Fuping and Xiaochuan, 2010). Therefore, TMT building should combine the principles of differentiation and diversity to select and appoint team members. When forming a team, it is necessary to consider not only the differences in structural characteristics, but also the diversity of the social relationship network characteristics of top management members. The negative moderating effect of TMT behavioral integration shows that for the acquisition of resources and the realization of entrepreneurial goals, mere information sharing is not sufficient because similar cognitive foundations will form similar problem-solving modes. Similarly, joint decision-making should be done while paying attention to the expression of individual opinions of members and respecting the differences between members, so as to avoid the phenomenon that effective opinions are not expressed and the decision-making body conforms to the crowd.

### Research Contributions

The study findings have theoretical value. Through quantitative analysis, this paper confirms the influence mechanism of resource bricolage on EO in start-ups. Resource constraint is the primary obstacle faced by start-ups, and even becomes an important reason for the low success rate and short duration of start-ups. Resource bricolage is an effective solution to overcome resource constraints of start-ups, and provides an effective way for start-ups to carry out entrepreneurial activities and create economic value. Bricolage can help entrepreneurs make optimal use of existing resources and circumvent resource constraints when start-ups cannot control all resources. From this point of view, this paper provides a new idea and feasible path for start-ups to overcome the resource dilemma, and the research conclusions enrich the research topics in the field of entrepreneurship.

The study findings also have practical implications. Entrepreneurs must be clearly aware of the importance of innovative strategy, proactive strategy and risk-taking strategy to the survival and development of new ventures Firstly, start-ups should pay attention to the market trend and grasp the market opportunity, in the new product creation and service mode and other aspects of new development. Secondly, start-ups should build a proactive strategic awareness to stay ahead of competitors from the start-up and management teams, and take a first-mover strategy to quickly capture the market and accumulate capital. Finally, to a certain extent, resource bricolage improves the utilization rate of resources, thus improving the risk bearing capacity of start-ups.

### Limitations

Limitations in the study suggest the following research avenues. Firstly, the study only studies the effect of resource bricolage strategy on entrepreneurial orientation during the start-up period, and the conclusions are only applicable to new ventures. Future research could explore the impact of resource bricolage strategy on entrepreneurial orientation in other growth stages. Secondly, the study takes only one country’s enterprises as samples. This suggests using international research in future to verify the conclusions with global data. Thirdly, one concern about the study is that readers may still be interested in the effect of entrepreneurial orientation on resource bricolage, although the study provides sufficient reasons to demonstrate the effect of resource bricolage on entrepreneurial orientation. It is a good thought process. People with low entrepreneurial orientation tend to take conservative action strategies in the face of insufficient resources, while people with high entrepreneurial orientation will reach their goals by bricolage, even though it is risky. In fact, our study pays more attention on the question of how start-ups thrive in resource-constrained situations. Resource bricolage can make full use of existing resources to improve the risk bearing capacity and enhance the innovation capacity of an enterprise. The study process is rigorous and the conclusions are credible. The future direction can be studied by studying the differences in the use of resource strategies among people with different degrees of entrepreneurial orientation. Fourthly, one question that needs to be explained is that in the study, the means of the key constructs are high and show little variation. When we set up the questionnaire to avoid ceiling effect and floor effect, it is necessary to explain the results to avoid misunderstanding. Firstly, we adopted mature scales and tested reliability and validity. Secondly, we carefully considered the language of the questionnaire, and further improved the questionnaire by communicating with three CEOs of start-ups to ensure that the interviewees fully understood the questions. Finally, a small number of questionnaires were collected in the way of pre-survey to test whether the design of the scale was reasonable. Based on this, we believe that the data results of this study are real and accurate, and there is no ceiling effect. We measure the extent to which the independent variable can explain the variation of the dependent variable by R-square value. The result shows that the R-square value is 0.513, indicating a good fitting degree.

## Data Availability Statement

The original contributions presented in the study are included in the article/supplementary material, further inquiries can be directed to the corresponding author.

## Author Contributions

PX guided and checked the topic selection and writing direction of the study. GR and SX are responsible for writing and revising the main part of the study. ZJ is responsible for data collection. All authors contributed to the article and approved the submitted version.

## Funding

This work was supported by National Natural Science Foundation of China, under programme “Research on regional innovation efficiency evaluation and cooperation object selection mechanism in sharing era” (no. 71701191) and University of Science and Technology of China initiated special fund project for research introduction of talents, “Research on the balance mechanism between business and public welfare social enterprises in Chinese context” (no. KY2160000003).

## Conflict of Interest

The authors declare that the research was conducted in the absence of any commercial or financial relationships that could be construed as a potential conflict of interest.

## Publisher’s Note

All claims expressed in this article are solely those of the authors and do not necessarily represent those of their affiliated organizations, or those of the publisher, the editors and the reviewers. Any product that may be evaluated in this article, or claim that may be made by its manufacturer, is not guaranteed or endorsed by the publisher.
